# Clinical and Molecular Spectrum of 1q21.1 Copy Number Variations: A Case Series

**DOI:** 10.3390/genes17101284

**Published:** 2026-10-15

**Authors:** Tuna Eren Esen, Lale Yilmaz Celik, Aydeniz Aydin Gumus, Pelin Ercoskun, Serdar Bozlak, Alper Gezdirici, Cuneyd Yavas, Ilknur Yilmaz

**Affiliations:** 1 Department of Medical Genetics, University of Health Sciences Basaksehir Cam and Sakura City Hospital, Istanbul 34460, Turkey; 2 Department of Pediatric Genetics, University of Health Sciences Basaksehir Cam and Sakura City Hospital, Istanbul 34480, Turkey; 3 Nevagen Genetic Diagnosis Center, Istanbul 34010, Turkey; 4 Department of Molecular Biology and Genetics, Faculty of Engineering and Natural Sciences, Biruni University, Istanbul 34460, Turkey; 5 Department of Molecular Biology and Genetics, Faculty of Engineering and Natural Sciences, Istanbul Atlas University, Istanbul 34203, Turkey

**Keywords:** 1q21.1, copy number variation, genotype–phenotype characterization, incomplete penetrance, neurodevelopmental disorders

## Abstract

Background: 1q21.1 copy number variations (CNVs) are recurrent rearrangements with a clinical spectrum, from neurodevelopmental disorders and congenital anomalies to apparently unaffected carrier status, reflecting incomplete penetrance and variable expressivity. Methods: Our chromosomal microarray (CytoScan HD) database was retrospectively screened for pathogenic or likely pathogenic 1q21.1 CNVs. Results: Twenty probands from 19 families (14 postnatal, 6 prenatal) carried 21 CNV events: 11 deletions (52.4%) and 10 duplications (47.6%). Of these, 16 (76.2%) were confined to the distal segment, 3 (14.3%) to the proximal segment, and 2 (9.5%) spanned both. One proband carried coexisting proximal duplication and distal deletion, both inherited from an apparently unaffected father. Of the 17 probands (85%) with parental testing, 7 (41.2%) had de novo and 10 (58.8%) inherited CNVs. Distal CNVs were mostly observed in probands with neurodevelopmental or cardiac phenotypes, whereas bilateral upper extremity anomalies were observed in both prenatally ascertained fetuses with isolated proximal deletions. Corpus callosum atrophy and choanal atresia were infrequently reported co-occurring findings, whereas prenatal intracranial hemorrhage was potentially novel; each occurred in one proband. Given the small proximal subgroup and differing ascertainment, these segment-associated trends are descriptive. Conclusions: This series highlights the relevance of segment localization and parental segregation data in interpreting 1q21.1 CNVs.

## 1. Introduction

Copy number variations (CNVs) constitute an important category of structural variants, and numerous recurrent CNV regions have been identified across different chromosomes [1]. One such region, 1q21.1, is among the relatively well-characterized recurrent rearrangement regions in the human genome [2]. Deletions and duplications in this region arise through non-allelic homologous recombination (NAHR) between flanking low-copy repeats (LCRs) [3]. Based on its genomic architecture, two subsegments are recognized: the proximal segment (BP1–BP3/BP2–BP3, chr1:145,686,999–146,048,495, GRCh38), which contains the *RBM8A* gene associated with thrombocytopenia-absent radius (TAR) syndrome, and the distal segment (BP3–BP4, chr1:147,105,904–147,917,509, GRCh38), which harbors *GJA5*.

Although proximal and distal 1q21.1 CNVs arise within the same complex segmental duplication architecture, they represent related but biologically distinct entities, and their clinical consequences differ according to the segment involved and whether the CNV is a deletion or a duplication [4,5,6]. The proximal *RBM8A*-containing TAR region has a different biological and clinical context. Proximal deletions are best established as the null allele in autosomal recessive TAR syndrome and are not sufficient by themselves to cause the disorder. Beyond TAR syndrome, alterations involving the proximal segment have been associated not only with structural anomalies but also with a range of neurodevelopmental features, particularly global developmental delay and intellectual disability [5]. Distal BP3–BP4 CNVs correspond to the recurrent 1q21.1 deletion and duplication syndromes. These distal CNVs are characterized by a more heterogeneous clinical spectrum; autism spectrum disorder (ASD), intellectual disability (ID), schizophrenia, epilepsy, congenital heart disease, and dysmorphic features are among the principal findings associated with this segment [7,8]. The “mirror phenotype,” characterized by microcephaly in deletion carriers and macrocephaly in duplication carriers, has been described as one of the most characteristic features of this region [9]. In particular, penetrance in distal 1q21.1 deletions and duplications has been reported to be incomplete and highly variable across studies, and marked phenotypic variability may be observed even among individuals carrying the same variant [10,11]. This variability is one of the main reasons why the clinical spectrum of 1q21.1 CNVs has not yet been fully delineated. In particular, the relative contribution of segment localization, rearrangement direction, and inheritance pattern to the resulting phenotype has not been systematically disentangled, limiting the precision of genotype–phenotype counseling.

Published case series on this region have made important contributions to the characterization of its clinical phenotype; however, previously unreported or rarely described findings continue to be identified through individual cases and targeted cohort studies [12,13]. In the present study, we report a case series of 20 probands. The main aims of this study were to describe the molecular characteristics of 1q21.1 CNVs, to explore segment-associated phenotypic trends, to document infrequently reported or potentially novel co-occurring clinical findings, and to contribute to the understanding of the clinical spectrum associated with incomplete penetrance and variable expressivity.

## 2. Materials and Methods

All diagnostic CMA data generated at our institution between January 2024 and 1 July 2025 were retrospectively screened for CNVs involving the 1q21.1 region, regardless of age, sex, or clinical indication. During this period, 2919 index cases underwent diagnostic CMA (749 prenatal and 2170 postnatal); parental samples analyzed for segregation were excluded from this number. The study cohort comprised the probands in whom an eligible 1q21.1 CNV was identified through this retrospective screening. Postnatal CMA had been performed for a broad range of clinical indications, predominantly neurodevelopmental disorders and congenital anomalies, whereas prenatal CMA had primarily been performed because of abnormalities detected by fetal ultrasonography or magnetic resonance imaging. In accordance with current quality standards, only CNVs—both gains and losses—with ≥20 probes/markers and classified as likely pathogenic or pathogenic were included in the study [14].

Based on these criteria, 20 probands from 19 families were identified, including two probands from a single family. The study was conducted in accordance with the principles of the Declaration of Helsinki and was approved by the Clinical Research Ethics Committee of Cam and Sakura City Hospital (Reference No: KAEK/21.05.2025.162, dated 26 July 2025). Written informed consent was obtained from all participants or their legal guardians. Cases were sequentially ascertained from the CMA database without further clinical pre-selection, and no eligible cases meeting the size and probe-count thresholds were excluded from the analysis.

The cohort comprised 20 probands from 19 families and 21 1q21.1 CNV events because one proband (P011) carried two coexisting CNVs. Unless otherwise specified, demographic and phenotypic data were analyzed at the proband level (n = 20), CNV type, size, and segment involvement at the CNV-event level (n = 21), and family-level data at the family level (n = 19). Inheritance analyses were based on the 17 probands with available parental testing. Because proximal and distal CNVs differ in gene content and established dosage sensitivity, CNVs confined to the distal region, CNVs confined to the proximal region, and CNVs spanning both regions were considered separately, and deletions and duplications were distinguished throughout the analyses. At the proband level, P011, who carried a proximal duplication coexisting with a distal deletion, was considered individually.

### 2.1. Chromosomal Microarray Analysis

All samples were analyzed using the CytoScan HD Array platform (Affymetrix/Thermo Fisher Scientific, Santa Clara, CA, USA). A total of 20 probands were evaluated, including 14 postnatal and 6 prenatal cases. Genomic DNA was extracted from peripheral blood leukocytes in postnatal cases and from amniocytes or chorionic villus samples in prenatal cases following the manufacturer’s instructions. Array data processing and CNV analysis were performed using Chromosome Analysis Suite (ChAS) (version4.4.1) software (Thermo Fisher Scientific) based on the GRCh38/hg38 reference genome. Exome or genome sequencing was not performed in any of the probands.

### 2.2. Clinical Data and Phenotype Definitions

Clinical data were collected retrospectively from the medical records, including clinical genetics evaluations and available neurodevelopmental, neuroimaging, cardiologic, and prenatal imaging records. Phenotypic features were categorized using predefined operational definitions. Global developmental delay was defined as significant delay in two or more developmental domains in children younger than 5 years. Intellectual disability was defined by deficits in intellectual and adaptive functioning with onset during the developmental period, as documented in the clinical records. Microcephaly and macrocephaly were defined as an occipitofrontal circumference more than 2 standard deviations below or above the age- and sex-specific mean, respectively. Short stature was defined as height more than 2 standard deviations below the age- and sex-specific mean. Anthropometric measurements were interpreted using age- and sex-appropriate reference standards used in routine clinical practice, including WHO and CDC growth references where applicable. Cardiac abnormalities were defined as structural congenital heart defects documented by postnatal echocardiography or prenatal fetal cardiac imaging. Because phenotypic data were collected retrospectively, the extent of assessment varied between individuals; features that were not documented were considered not assessed rather than absent.

### 2.3. CNV Interpretation and Classification

Detected CNVs were interpreted through integrated evaluation of genomic localization, size, gene content, inheritance status, and available literature and database information. CNV classification was performed in accordance with the standards and guidelines recommended by the American College of Medical Genetics and Genomics (ACMG) and the Clinical Genome Resource (ClinGen) [15]. Each CNV, including the two CNVs in P011, was classified independently using the ACMG/ClinGen point-based scoring framework; evidence codes, points, and total scores are provided in Supplementary Table S1. The following databases were used to support variant interpretation: Database of Genomic Variants (DGV), ClinVar, DECIPHER, and the ClinGen Dosage Sensitivity Map (accessed 25 April 2026) [16,17]. Additional CNVs detected outside the 1q21.1 region were also reviewed, and their clinical classifications were recorded.

Cohort ascertainment was based on the diagnostic laboratory classifications available at the time of testing; the event-level classifications presented here reflect an independent re-evaluation and may therefore differ from the original laboratory classification for individual CNVs. The proximal duplication in P011, classified as a variant of uncertain significance, was retained because it coexisted with a pathogenic distal deletion. Distal deletions were scored with criterion 2A (ISCA-37421, haploinsufficiency score 3). Because the distal region has a triplosensitivity score of 2, criterion 2A was not applied to duplications, which were scored using case–control evidence (4L, 4M). The proximal deletions in P003 and P008 were interpreted as null alleles of *RBM8A* in the context of autosomal recessive TAR syndrome. Available parental segregation information was incorporated into the independent re-evaluation when applicable under the ACMG/ClinGen scoring framework. Where inheritance contributed directly to the quantitative score, the corresponding evidence code is reported in Supplementary Table S1.

### 2.4. Parental Segregation Analysis

Parental segregation analysis was performed in 17 of 20 probands (85%); parental samples were unavailable for three probands. Parental samples analyzed at our institution were tested using the same platform and methodology; in P007, paternal CMA had been performed at an external center and was reported as normal. The resulting data were used to determine the inheritance pattern of the identified CNVs and to support clinical interpretation. All transmitting parents underwent clinical genetics evaluation. Parents without clinically apparent abnormalities on this evaluation are described as apparently unaffected. This designation does not exclude subtle neurodevelopmental, behavioral, psychiatric, cardiac, or other manifestations that might require targeted standardized assessment.

## 3. Results

A total of 20 probands from 19 families were included in the study. Of the cases, 12 were male (60%) and 8 were female (40%); 14 were evaluated postnatally and 6 prenatally. Two affected siblings from the same family were included in the cohort. In addition, one proband carried two coexisting 1q21.1 CNVs, a proximal duplication and a distal deletion.

### 3.1. Segment-Based Molecular Landscape of 1q21.1 CNVs

#### 3.1.1. CNV Types and Sizes

**Figure 1:**
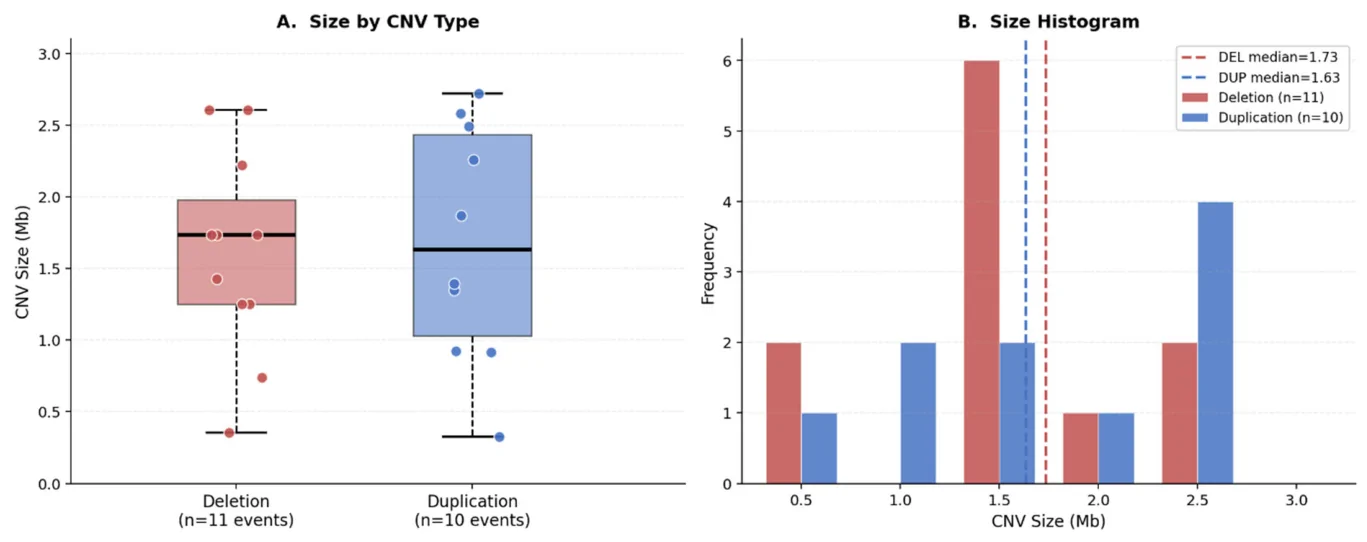
CNV Size Distribution. (**A**) Graph showing CNV sizes in the deletion and duplication groups. The upper and lower borders of the box represent the 75th and 25th percentiles, respectively, and the horizontal line indicates the median; dots represent individual CNV events. (**B**) Histogram summarizing the size distribution of all CNV events; dashed vertical lines indicate the median sizes of the deletion (red, median: 1.733 Mb) and duplication (blue, median: 1.633 Mb) groups.

Of the 21 CNV events identified in the 20 probands, 11 (52.4%) were deletions and 10 (47.6%) were duplications. Deletion sizes ranged from 0.355 to 2.606 Mb (median: 1.733 Mb), whereas duplication sizes ranged from 0.325 to 2.720 Mb (median: 1.633 Mb) (Figure 1).

#### 3.1.2. Segment Involvement

Of the 21 CNV events, 18 (85.7%) involved the distal 1q21.1 segment: 16 (76.2%) were confined to the distal segment and 2 (9.5%) spanned both the proximal and distal segments. The remaining 3 events (14.3%) were confined to the proximal segment. A proband-level summary of segment classification, CNV type, size, inheritance, and primary phenotype for all cases is provided in Table 1, with full demographic, molecular, and clinical details available in Supplementary Table S1.

**Figure 2:**
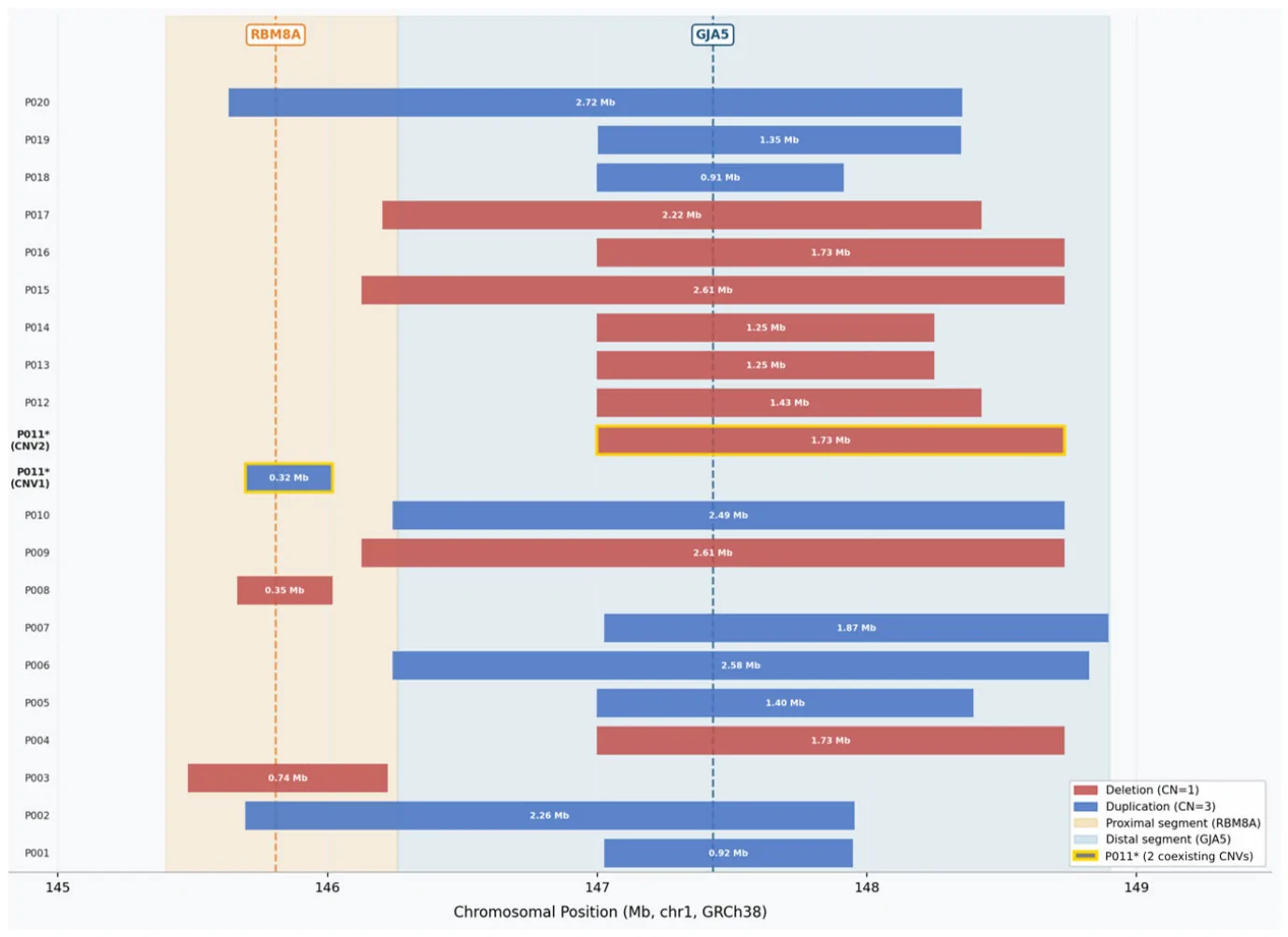
Genomic Map of 1q21.1 CNVs. The orange background represents the proximal segment (BP1–BP3/BP2–BP3; RBM8A gene region), whereas the light blue background represents the distal segment (BP3–BP4; GJA5 gene region); shading is approximate, and the segment assignment of each CNV was based on its overlap with the ClinGen-curated regions (ISCA-37428 and ISCA-37421) given in Supplementary Table S1. P011 *, highlighted with a yellow box, indicates the proband with two coexisting 1q21.1 CNVs (a proximal duplication and a distal deletion), both inherited from the father (* indicates that patient have an extra CNV).

In the distal group, 9 deletions and 7 duplications were identified, whereas the proximal group included 2 deletions and 1 duplication; the two CNVs spanning both proximal and distal regions were duplications. In one proband (P011), a proximal duplication (0.325 Mb) and a distal deletion (1.733 Mb) were detected; both were inherited from the father, who also carried a proximal duplication and a distal deletion. Chromosomal microarray analysis does not determine the structure or phase of these CNVs. This case was the only one in the series in which two distinct 1q21.1 CNVs were detected in the same individual (Figure 2).

#### 3.1.3. Additional CNVs

In addition to the 1q21.1 CNVs, autosomal CNVs outside the 1q21.1 region were detected in four probands: a 0.294 Mb deletion at 6q14.1 in P001, a 0.673 Mb deletion at 20q13.13 in P015, a 0.254 Mb deletion at 4q12 and a 0.651 Mb duplication at 6q16.1 in P017, and a 0.547 Mb deletion at 11p12 in P020 (Supplementary Table S1). Small CNVs involving the sex chromosomes were detected in several probands, some of which were also present in a parent. All additional CNVs were classified as benign or likely benign and were not considered a plausible primary explanation for the clinical phenotypes. Exome or genome sequencing was not performed in any proband.

Because none of the confirmed additional CNVs was classified as a variant of uncertain significance, likely pathogenic, the CMA findings do not support a blended CNV phenotype. Hemihypertrophy and Wilms tumor in P017 were not attributed to the 1q21.1 CNV, and an independent cause cannot be excluded.

### 3.2. Inheritance Pattern

**Figure 3:**
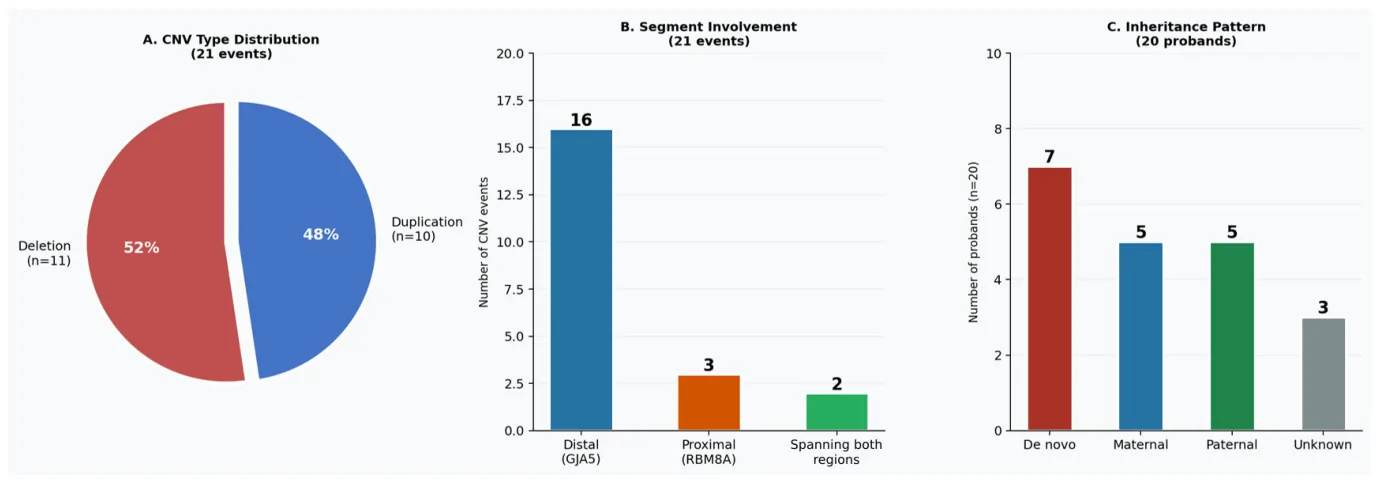
Molecular Characteristics of 1q21.1 CNVs. (**A**) Distribution of CNV types among 21 CNV events. (**B**) Segment distribution among 21 CNV events. (**C**) Inheritance pattern at the proband level (n = 20); the “Unknown” category represents the three probands without parental testing, and inheritance percentages reported in the text are calculated among the 17 probands with parental testing. P011 is counted once in panel C because both of her CNVs were inherited from the father.

Parental segregation analysis was performed in 17 of 20 probands (85%). Among the 17 probands with parental testing, the CNV was inherited in 10 (58.8%) and arose de novo in 7 (41.2%). Of the 10 probands with an inherited CNV, 5 inherited the CNV maternally and 5 paternally (50% each). Inheritance at the proband level is shown in Figure 3C.

### 3.3. Clinical Phenotypes and Segment-Associated Observations

#### 3.3.1. Neurodevelopmental Phenotypes

ASD was recorded as the most frequent primary clinical finding in the postnatal group and was identified in 6 probands (P001, P005, P007, P010, P013, and P014). All of these probands carried distal segment CNVs; four had duplications and two had deletions. The two sisters from family F013 (P013, aged 14 years; P014, aged 7 years) carried the same paternally inherited 1.251 Mb distal deletion, and both had been diagnosed with ASD.

GDD was recorded as the primary clinical finding in two probands. In P006 (13-year-old male, maternal distal duplication, 2.582 Mb), ADHD, strabismus, and diffuse corpus callosum atrophy on cranial MRI were recorded in addition to GDD. P018 (10-year-old male, distal duplication, 0.914 Mb) was evaluated for GDD and epilepsy. In P020 (3-year-old female, duplication spanning both proximal and distal regions, 2.720 Mb, de novo), speech and language delay was recorded together with epilepsy, scoliosis, and bilateral extrarenal renal pelvis dilatation. In P005 (10-year-old male, distal duplication, 1.396 Mb, de novo), ataxia and mild myogenic changes in the distal upper and lower extremities on EMG were recorded in addition to ASD.

#### 3.3.2. Cardiac Phenotypes

Congenital heart disease was identified in 3 probands, all of whom carried distal segment CNVs. In P004 (newborn female, paternal distal deletion, 1.733 Mb), echocardiography revealed complete atrioventricular septal defect (AVSD), a large ostium primum-type atrial septal defect, a small ventricular septal defect (VSD), atrioventricular valve insufficiency, and aortic valve insufficiency. In P012 (16 gestational weeks, male fetus, de novo distal deletion, 1.427 Mb), a single umbilical artery, VSD, a prominent philtrum, retrognathia, and a posterior fossa cystic lesion were identified. P019 (19 gestational weeks, male fetus, de novo distal duplication, 1.348 Mb) was evaluated for a conotruncal cardiac anomaly characterized by marked left axis deviation, hypoplastic pulmonary artery, 50% abnormally positioned aorta, and subaortic VSD.

#### 3.3.3. Prenatal Phenotypes

The six prenatally diagnosed probands are presented here as a separate descriptive subgroup; abnormal fetal imaging findings were identified in all six. Bilateral upper extremity anomalies were detected in the two prenatal cases carrying proximal segment deletions (P003 and P008). In P003 (21 gestational weeks, female fetus, proximal deletion, 0.741 Mb; parental analysis could not be performed), bilateral humeral bowing and shortening, bilateral elbow and hand flexion contractures, and bilateral radial ray anomalies were identified. P008 (21 gestational weeks, male fetus, proximal deletion, 0.355 Mb, maternal inheritance) was evaluated for bilateral flexion contractures and bilateral radial ray anomalies; the mother was found to carry a larger proximal deletion (0.537 Mb) and was apparently unaffected, with a normal platelet count. Both fetuses were live-born. Postnatally, the radial ray anomaly corresponded to bilateral absence of the radius with thumbs present, and a history of thrombocytopenia was noted in the postnatal records, although numerical platelet values were not available. Because the available DNA quantity was insufficient for sequencing, the second *RBM8A* allele, including known regulatory variants, could not be investigated. In P002 (23 gestational weeks, male fetus, paternal inheritance, 2.259 Mb), who carried a duplication spanning both the proximal and distal regions, hypoplastic nasal bone below the 5th percentile (5.1 mm) was identified. Among the three prenatal cases with distal segment CNVs, P012 and P019 are discussed in detail under Section 3.3.2 because both presented with cardiac involvement. P012 was a 16-gestational-week fetus with a de novo distal deletion of 1.427 Mb and cardiac findings accompanied by dysmorphic features, whereas P019 was a 19-gestational-week fetus with a de novo distal duplication of 1.348 Mb and a conotruncal cardiac anomaly. In P015, a 12-gestational-week fetus with a de novo distal deletion of 2.606 Mb, fetal MRI demonstrated hemorrhagic findings in the right caudate head and lentiform nucleus, cortical atrophy, and asymmetric lateral ventricular enlargement.

The pregnancies of P012 and P019 were terminated after major structural abnormalities were identified on prenatal imaging. In P015, intrauterine fetal death occurred after the detection of fetal cerebral hemorrhagic findings. These outcomes are reported descriptively and should not be interpreted as consequences of the 1q21.1 CNVs themselves. P002, P003, and P008 were live-born; apart from the available postnatal information for P003 and P008, systematic postnatal follow-up data were not available, and postnatal phenotypic evolution could not be assessed.

#### 3.3.4. Segment-Associated Observations

**Figure 4:**
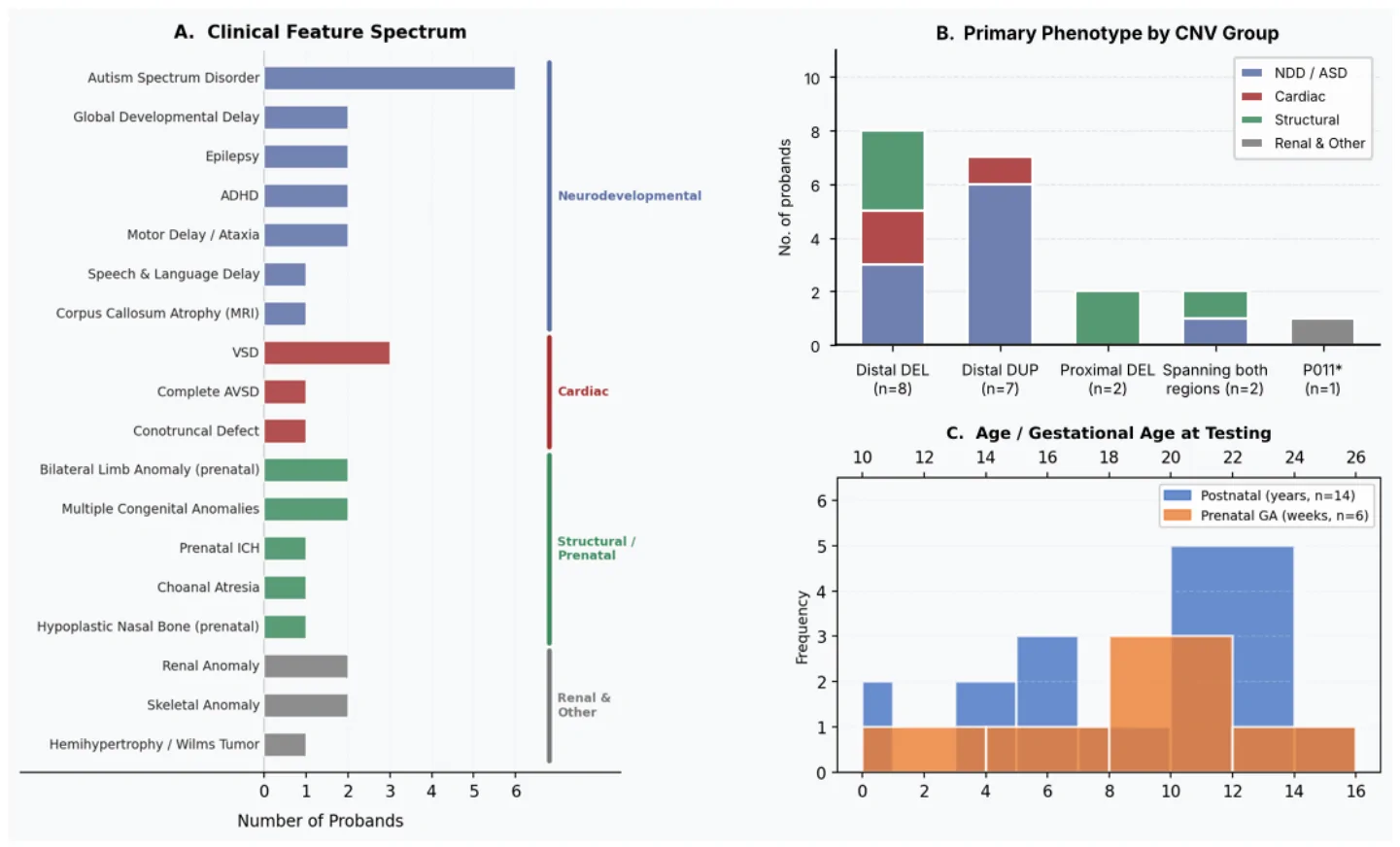
Clinical Profile of 1q21.1 CNVs. (**A**) Distribution of the clinical spectrum. (**B**) Primary phenotype by CNV group at the proband level (n = 20): distal deletion (n = 8), distal duplication (n = 7), proximal deletion (n = 2), duplications spanning both regions (n = 2), and P011, who carried a proximal duplication coexisting with a distal deletion (n = 1). (**C**) Distribution of age or gestational age at testing. ASD: autism spectrum disorder; NDD: neurodevelopmental disorder; GDD: global developmental delay; ADHD: attention-deficit/hyperactivity disorder; VSD: ventricular septal defect; AVSD: atrioventricular septal defect; ICH: intracranial hemorrhage; MCA: multiple congenital anomalies; GA: gestational age (* indicates that patient have an extra CNV).

Because proximal *RBM8A*-containing CNVs and distal recurrent 1q21.1 CNVs represent biologically distinct entities, their clinical observations are summarized separately rather than as a single phenotypic continuum (Figure 4). Among the 12 postnatally evaluated probands with distal-only CNVs, the primary phenotype was neurodevelopmental in nine, and all three probands with congenital heart disease (one postnatal and two prenatal) carried distal CNVs. The proximal subgroup was much smaller: isolated proximal deletions were identified only in P003 and P008, both of whom were ascertained prenatally because of bilateral upper-limb abnormalities. No proband carried an isolated proximal duplication; the proximal duplication in P011 coexisted with a distal deletion. The two duplications spanning both proximal and distal regions were identified in a fetus with a hypoplastic nasal bone (P002) and in a child with speech and language delay and epilepsy (P020). Given the small proximal subgroup, the exclusively prenatal ascertainment of the isolated proximal deletions, and the absence of an isolated proximal duplication, these data do not permit a direct phenotypic comparison between proximal and distal CNVs or establish a proximal genotype–phenotype pattern.

## 4. Discussion

1q21.1 CNVs are among the genomic disorders characterized by low penetrance, variable expressivity, and broad phenotypic diversity [11]. Rearrangements in this region may be associated with heterogeneous clinical manifestations ranging from neurodevelopmental disorders and neurobehavioral phenotypes to congenital heart disease and structural and dysmorphic findings [4,6]. This variability has been reported to differ according to segment localization and whether the CNV is a deletion or a duplication [4,5,6]. The OMIM entries “Chromosome 1q21.1 deletion syndrome” and “Chromosome 1q21.1 duplication syndrome” refer to the recurrent distal CNVs, whereas TAR syndrome is a distinct disorder associated with the proximal *RBM8A*-containing region. Proximal CNVs and larger CNVs spanning both regions were therefore interpreted separately in this study.

Deletions and duplications involving the proximal 1q21.1 region, particularly around the TAR region (BP1–BP3/BP2–BP3), have been defined as a susceptibility locus for both classic TAR syndrome and neurodevelopmental and multiple congenital anomalies [5]. Proximal 1q21.1 deletions have been associated with TAR syndrome, skeletal anomalies (radius/humerus hypoplasia, short curved ribs), and brainstem hypoplasia, whereas proximal duplications have been reported in association with developmental delay, intellectual disability, autism spectrum disorder, behavioral problems, and dysmorphic features [18].

Both proximal deletions in this cohort (P003 and P008) include *RBM8A* and overlap the ClinGen-curated proximal TAR region (ISCA-37428); their exact coordinates and breakpoint classes are provided in Supplementary Table S1. In the present cohort, the clinical features of P003 and P008 were considered potentially compatible with TAR syndrome, including bilateral absence of the radius with preserved thumbs and a history of thrombocytopenia. However, the proximal deletion represents an *RBM8A* null allele that is not sufficient by itself to explain TAR syndrome; because the second *RBM8A* allele could not be investigated, TAR syndrome could not be molecularly confirmed. The mother of P008, who carries the proximal deletion, was apparently unaffected on clinical genetics evaluation and had a normal platelet count, consistent with the autosomal recessive mechanism of TAR syndrome.

Distal 1q21.1 deletions and duplications are associated with developmental delay, cognitive dysfunction, motor skill and tone abnormalities (fine and gross motor impairment, hypotonia), speech/articulation problems, dysmorphic facial features, and various congenital anomalies (particularly involving the heart and urinary system; prenatal ultrasonographic findings such as increased NT, cardiac defects, skeletal anomalies, and ventriculomegaly) [13,19]. At the neurodevelopmental and neurobehavioral level, both CNV types have been reported in association with autism spectrum disorder, learning difficulties, epilepsy/seizures, and other behavioral problems. Table 2 provides a qualitative overview of the phenotypic features reported in selected published studies and is not intended for direct comparison of phenotype frequencies.

In our study, bilateral upper extremity anomalies, humeral shortening, and flexion contractures were identified in the two cases carrying proximal segment deletions, in line with previous reports [5,24]; however, with only two such cases, both referred because of limb anomalies, they cannot be taken as evidence of a segment-specific association. Distal segment CNVs were detected together with neurodevelopmental disorders, congenital heart disease, renal anomalies, and multiple congenital anomalies. Although prenatal findings have been reported in previous 1q21.1 CNV cohorts, the present series provides additional case-level prenatal data, including fetal presentations according to the segment involved and pregnancy outcomes. Bilateral upper extremity anomalies were observed in both prenatally ascertained fetuses with isolated proximal deletions, whereas distal CNVs were observed together with cardiac and central nervous system findings. Given the small number of prenatal cases, these findings should be interpreted descriptively, but they may contribute to prenatal counseling and phenotype delineation. In this series, ASD was identified predominantly in duplication carriers. Head circumference measurements were available for all 14 postnatal probands and were within the normal range in all of them. Accordingly, the classical deletion-associated microcephaly and duplication-associated macrocephaly “mirror phenotype” was not observed in this cohort.

Diffuse corpus callosum atrophy in P006, prenatal intracranial hemorrhage in P015, and choanal atresia in P016 were each observed in a single individual and should not be interpreted as established manifestations of 1q21.1 CNVs. Reduced corpus callosum volume has been reported in 3 of 13 carriers of the 1q21.1 duplication [10], and agenesis of the corpus callosum in an individual with a proximal deletion [11]; the finding in P006, who carried a distal duplication, is therefore best regarded as an infrequently reported co-occurring finding. Choanal atresia has been described in an individual with a 1.7-Mb deletion of the proximal 1q21.1 region [24], whereas P016 carried a distal deletion; this finding is therefore also considered infrequently reported in carriers of 1q21.1 CNVs. To our knowledge, prenatal intracranial hemorrhage has not been previously reported in association with 1q21.1 CNVs, and this finding in P015 is considered a potentially novel co-occurring observation. Alternative explanations cannot be excluded. No additional CNV was identified in P006, whereas P015 carried a likely benign 20q13.13 deletion and P016 carried only a 16-kb loss in pseudoautosomal region 2, classified as benign or likely benign. The evaluation for alternative causes of fetal intracranial hemorrhage in P015 was incomplete; therefore, a prenatal complication or another etiology cannot be excluded. In P016, CHARGE syndrome, an important genetic differential diagnosis of choanal atresia, was considered clinically but was not supported by the available findings; *CHD7* testing was not performed. Furthermore, exome or genome sequencing was not performed in any of these patients, and pathogenic sequence variants in other genes therefore cannot be excluded. These observations are hypothesis-generating and require confirmation in independent cases or larger cohorts.

P011 carried a coexisting proximal duplication and distal deletion, both inherited from her apparently unaffected father. Chromosomal microarray analysis does not determine the physical structure or phase of these CNVs; therefore, paternal inheritance of both CNVs does not establish that they occur in cis, and their structure and phase remain undetermined. Establishing their structural relationship and phase would require additional molecular studies, which were not performed in this case. Renal ectopia and short stature in the proband do not fully overlap with the classical proximal duplication or distal deletion phenotype. To the best of our knowledge, no case of a coexisting proximal duplication and distal deletion in the same individual has been reported in the literature.

Incomplete penetrance and intrafamilial phenotypic variability are among the notable clinical findings in this series. For recurrent distal 1q21.1 CNVs, Rosenfeld et al. estimated the probability of an abnormal pediatric phenotype at 36.9% (95% CI, 23.0–55.0%) for deletions and 29.1% (95% CI, 16.9–46.8%) for duplications [25]. These estimates apply specifically to recurrent distal CNVs and should not be generalized to proximal 1q21.1 rearrangements or interpreted as risks for any single phenotype. Because the present series was ascertained through clinically referred probands, it does not permit an independent estimate of penetrance. Among the 17 probands with parental testing, the CNV was inherited in 10; all transmitting parents were apparently unaffected on clinical genetics evaluation. The findings in family F004 provided the clearest example of this relationship: while the proband presented with severe congenital heart disease involving complete AVSD and multiple valvular abnormalities, the father and brother carrying the same paternal deletion were apparently unaffected. In family F013, two siblings carrying the same paternal deletion had ASD, whereas the carrier father was apparently unaffected; this pattern is consistent with the intrafamilial variability of expressivity described in neurodevelopmental CNV syndromes [13,21]. Within the available retrospective records, no major phenotypic difference between the two sisters was documented; this family therefore illustrates intrafamilial variability primarily between the affected siblings and their transmitting father rather than marked phenotypic discordance between the sisters.

In seven of the nine families with an inherited CNV, the array-reported coordinates of the parental and proband CNVs differed (Supplementary Table S1). A germline CNV transmitted from a parent is expected to share the same biological breakpoints. The differing boundaries were located within the segmental duplication-rich regions flanking the 1q21.1 breakpoints, where probe coverage is limited. These differences are therefore most likely attributable to probe distribution and segmentation in these regions rather than to true breakpoint differences. Because orthogonal confirmation was not performed, inheritance in these families is based on CMA findings alone. Clinically, a difference in array-reported size between parental and proband CNVs is not sufficient grounds to exclude inheritance. In regions with repetitive genomic architecture such as 1q21.1, this issue is important for the accurate interpretation of inheritance patterns.

The present findings indicate that, together with the segment involved, incomplete penetrance and variable expressivity are major considerations in the clinical interpretation of 1q21.1 CNVs. In our series, the presence of apparently unaffected carrier parents in inherited cases and the observation of different degrees of clinical severity within the same segment support the limited predictive value of phenotype [26]. Therefore, the interpretation of cases with 1q21.1 CNVs should not be based solely on the presence of the CNV itself, but should instead incorporate segment content, the direction of the rearrangement, inheritance pattern, and accompanying clinical findings. This integrated approach may improve the accuracy of genetic counseling and clinical follow-up in both prenatal and postnatal settings.

This study has several limitations. The retrospective, single-center design may limit generalizability. In addition, the prenatal subgroup was small (n = 6), restricting conclusions to a descriptive level. Importantly, the proximal subgroup was too small and differently ascertained to permit independent phenotypic characterization or direct comparison with the distal recurrent CNV group. Only two probands carried isolated proximal deletions, and no isolated proximal duplication was identified. Moreover, neurodevelopmental features such as ASD, ID, speech delay, and epilepsy cannot be assessed prenatally. Because the proximal and distal intervals are also biologically distinct, these factors preclude a direct proximal-versus-distal genotype–phenotype comparison in this cohort. More broadly, although inclusion in the present study was based on the identification of an eligible 1q21.1 CNV rather than on clinical indication, the prenatal and postnatal groups originated from different referral pathways. Prenatal cases had undergone CMA primarily because of abnormalities detected on fetal imaging, whereas postnatal cases had undergone CMA predominantly because of neurodevelopmental disorders or congenital anomalies. The cohort therefore represents a clinically referred population and is subject to referral and ascertainment bias; the observed phenotype distributions should not be generalized to unselected carriers of 1q21.1 CNVs. Phenotypic data were also collected retrospectively from medical records, and the extent of phenotypic assessment varied between individuals. Moreover, chromosomal microarray analysis cannot determine the structure or phase of the coexisting proximal duplication and distal deletion in P011, and no additional molecular studies were performed to establish their structural relationship or phase. Inheritance was determined by CMA, and orthogonal confirmation of the parental CNVs was not performed. In P003 and P008, numerical platelet values were not available, and the available DNA quantity was insufficient for *RBM8A* sequencing; TAR syndrome could therefore not be molecularly confirmed. In addition, exome or genome sequencing was not performed; therefore, sequence-level genetic contributors to the observed phenotypes, including the infrequently reported or potentially novel co-occurring findings, cannot be excluded. The etiologic evaluation of prenatal intracranial hemorrhage in P015 was also incomplete, limiting causal interpretation of this finding. Finally, parental samples were unavailable for three probands, and systematic postnatal follow-up was not available for the live-born prenatally diagnosed probands (P002, P003, and P008). Despite these limitations, the inclusion of both prenatal and postnatal cases and the availability of parental segregation data for most probands provide case-level information that may inform larger genotype–phenotype studies.

## 5. Conclusions

In conclusion, this case series contributes to the clinical and molecular documentation of 1q21.1 CNVs by integrating prenatal and postnatal presentations, inheritance data, and segment-level observations. The findings support the broad phenotypic variability and incomplete penetrance characteristic of this locus and underscore the importance of parental segregation analysis for genetic counseling. The segment-associated trends observed in this cohort, including upper extremity anomalies in two prenatally ascertained fetuses with proximal deletions and the predominance of neurodevelopmental and cardiac presentations among distal CNV carriers, are descriptive and do not establish a segment-dependent genotype–phenotype relationship. This series does not allow conclusions about the phenotypes associated with proximal CNVs. Proximal *RBM8A*-containing rearrangements should therefore be interpreted separately from the distal recurrent 1q21.1 deletion and duplication syndromes. Corpus callosum atrophy and choanal atresia, each observed in a single proband, are infrequently reported co-occurring findings, whereas prenatal intracranial hemorrhage, observed in one fetus, is a potentially novel co-occurring finding; none should be considered attributable to 1q21.1 CNVs without confirmation in independent cases or larger cohorts. Future multi-center studies with larger prenatal cohorts and long-term postnatal follow-up will be essential to further refine penetrance estimates and to validate the phenotypic observations described here.

## Figures and Tables

**Table 1:** Proband-Level Summary of 1q21.1 CNV Characteristics.

Proband	Segment	CNV Type	Size (Mb)	Inheritance	Primary Phenotype
P001	Distal	Duplication	0.922	Maternal	Autism spectrum disorder
P002	Spanning both regions	Duplication	2.259	Paternal	Hypoplastic nasal bone (prenatal)
P003	Proximal	Deletion	0.741	Not performed	Bilateral upper extremity anomalies (prenatal)
P004	Distal	Deletion	1.733	Paternal	Complex congenital heart disease
P005	Distal	Duplication	1.396	De novo	Autism spectrum disorder, ataxia
P006	Distal	Duplication	2.582	Maternal	Global developmental delay, corpus callosum atrophy
P007	Distal	Duplication	1.870	De novo	Autism spectrum disorder
P008	Proximal	Deletion	0.355	Maternal	Bilateral upper extremity anomalies (prenatal)
P009	Distal	Deletion	2.606	Maternal	Multiple congenital anomalies
P010	Distal	Duplication	2.490	De novo	Autism spectrum disorder
P011 *	Proximal; distal	Duplication + Deletion	0.325 + 1.733	Paternal (both)	Renal ectopia, short stature
P012	Distal	Deletion	1.427	De novo	Congenital heart disease (prenatal)
P013	Distal	Deletion	1.251	Paternal	Autism spectrum disorder
P014	Distal	Deletion	1.251	Paternal	Autism spectrum disorder
P015	Distal	Deletion	2.606	De novo	Prenatal intracranial hemorrhage
P016	Distal	Deletion	1.733	Maternal	Choanal atresia, multiple congenital anomalies
P017	Distal	Deletion	2.223	Not performed	Attention-deficit/hyperactivity disorder
P018	Distal	Duplication	0.914	Not performed	Global developmental delay, epilepsy
P019	Distal	Duplication	1.348	De novo	Conotruncal cardiac anomaly (prenatal)
P020	Spanning both regions	Duplication	2.720	De novo	Speech/language delay, epilepsy

CNV, copy number variation. * P011 carried two coexisting 1q21.1 CNVs, a proximal duplication and a distal deletion, both inherited from the father. Full demographic, molecular, and clinical details for all probands and available family members are provided in Supplementary Table S1.

**Table 2:** Qualitative overview of phenotypic features reported in selected studies of 1q21.1 CNVs, with quantitative data from the present cohort.

Phenotypic Feature	Mefford et al., 2008 [9]	Brunetti-Pierri et al., 2008 [4]	Bernier et al., 2016 [10]	Pang et al., 2020 [11]	Linden et al., 2021 [20]	Edwards et al., 2021 [13]	Bourgois et al., 2024 [21]	Yue et al., 2023 [22]; Liu et al., 2025 [23]	Present Study
*Cohort (CNV Composition)*	*34 (25 del, 9 dup)*	*36 (21 del, 15 dup)*	*38 (19 del, 19 dup)*	*36 (20 del, 16 dup)*	*123 (68 del, 55 dup)*	*47 distal del*	*34 (8 del, 24 dup, 2 trip)*	*Yue: 26 (11 del, 15 dup), Liu: 20 (12 del, 8 dup); Prenatal*	*20 (21 Events: 11 del, 10 dup)*
**Neurodevelopmental phenotypes**									
Autism spectrum disorder	Reported	Reported	Reported	Reported	Reported	Reported	Reported	NR	6 ‡
Global developmental delay/ID	Reported	Reported	Reported	Reported	Reported	Reported	Reported	NR	2 ‡
ADHD/behavioral problems	Reported	NR	Reported	Reported	Reported	Reported	Reported	NR	2 ‡
Epilepsy/seizures	Reported	Reported	Reported	Reported	Reported	Reported	Reported	NR	2 ‡
Ataxia	NR	NR	NR	NR	NR	NR	NR	NR	1 ‡
EMG myogenic changes	NR	NR	NR	NR	NR	NR	NR	NR	1 ‡
**Neuroimaging findings**									
Microcephaly/ macrocephaly	Reported	Reported	Reported	Reported	Reported	Reported	Reported	NR	0/14
Corpus callosum abnormality (MRI)	NR	NR	Reported	Reported	NR	NR	NR	NR	1 ‡
Cerebral ventriculomegaly (prenatal)	NR	NR	NR	NR	NR	NR	NR	Reported	1/6
Prenatal intracranial hemorrhage	NR	NR	NR	NR	NR	NR	NR	NR	1/6
**Cardiac phenotypes**									
Congenital heart defect (any)	Reported	Reported	Reported	Reported	NR	Reported	Reported	Reported	3 ‡
VSD/atrial septal defect	Reported	Reported	Reported	Reported	NR	Reported	Reported	Reported	3 ‡
Complete AVSD	NR	NR	NR	NR	NR	Reported	NR	NR	1 ‡
Conotruncal defects (TOF/TGA)	Reported	NR	NR	NR	NR	Reported	Reported	Reported	1 ‡
**Craniofacial features**									
Dysmorphic facial features	Reported	Reported	Reported	Reported	NR	Reported	Reported	Reported	3 ‡
Retrognathia/prominent philtrum	NR	Reported	NR	Reported	NR	Reported	Reported	Reported	2 ‡
Low-set or dysplastic ears	Reported	Reported	NR	Reported	NR	Reported	Reported	NR	2 ‡
Choanal atresia †	NR	NR	NR	NR	NR	NR	NR	NR	1 ‡
**Skeletal and extremity anomalies**									
Radial ray anomaly/TAR-like	Reported	NR	NR	Reported	NR	NR	NR	Reported	2 ‡
Bilateral flexion contractures	NR	NR	NR	NR	NR	NR	NR	Reported	2 ‡
Humeral shortening or bowing	NR	NR	NR	NR	NR	NR	NR	NR	1 ‡
Scoliosis	NR	NR	Reported	NR	NR	Reported	Reported	NR	1 ‡
Short stature	Reported	Reported	Reported	Reported	NR	Reported	Reported	NR	1/14
**Renal and urogenital anomalies**									
Renal anomaly	Reported	Reported	NR	Reported	NR	Reported	Reported	Reported	2 ‡
Renal ectopia	NR	NR	NR	NR	NR	NR	NR	NR	1 ‡
Hypospadias/anorectal malformation	NR	Reported	NR	Reported	NR	Reported	Reported	NR	1 ‡
**Prenatal phenotypic features**									
Abnormal prenatal ultrasonography (any)	NR	Reported	NR	Reported	NR	NR	Reported	Reported	6/6
Hypoplastic nasal bone	NR	NR	NR	NR	NR	NR	NR	Reported	1/6
Prenatal limb anomaly	NR	NR	NR	Reported	NR	NR	NR	Reported	2/6
Prenatal cardiac anomaly	NR	Reported	NR	Reported	NR	NR	Reported	Reported	2/6
Prenatal CNS anomaly	NR	NR	NR	NR	NR	NR	NR	Reported	2/6

For published studies, “Reported” indicates that the feature was described in at least one individual, and NR indicates that it was not reported or was not systematically assessed. These columns indicate only whether a feature has been reported; they do not represent phenotype frequencies and should not be interpreted as a comparison of phenotype prevalence across studies, because the studies differ substantially in cohort size, CNV type and segment composition, ascertainment, and depth of phenotypic assessment. One study focused on psychiatric and neurodevelopmental phenotypes [20], whereas the other two represented prenatal cohorts with limited opportunity for postnatal phenotypic assessment [22,24]. For the present study, n/N is reported only when the number of probands in whom the feature was assessed could be established reliably from the retrospective records. Head circumference and height were available for all 14 postnatal probands, and prenatal imaging findings were assessed in all 6 prenatal probands. ‡ For neurodevelopmental, neuroimaging, cardiac, craniofacial, skeletal, and renal/urogenital features for which a reliable feature-specific denominator could not be established, only the number of affected probands is shown. Features documented only in the present study were observed in single individuals, and coincidental co-occurrence cannot be excluded. Upper-limb findings (radial ray anomalies, flexion contractures, and humeral shortening or bowing, including the corresponding prenatal limb anomalies) were observed only in the two fetuses with isolated proximal deletions (P003 and P008), and renal ectopia and short stature only in P011, who carried a proximal duplication coexisting with a distal deletion. † Choanal atresia has been described in a single individual with a proximal 1q21.1 deletion [24]. ADHD, attention-deficit/hyperactivity disorder; AVSD, atrioventricular septal defect; CNS, central nervous system; CNV, copy number variation; del, deletion; dup, duplication; trip, triplication; EMG, electromyography; ID, intellectual disability; MRI, magnetic resonance imaging; NR, not reported; TAR, thrombocytopenia-absent radius; TGA, transposition of the great arteries; TOF, tetralogy of Fallot; VSD, ventricular septal defect.

## Data Availability

The original contributions presented in this study are included in the article material. Further inquiries can be directed to the corresponding author.

## Supplementary Materials

The following supporting information can be downloaded at https://www.mdpi.com/article/10.3390/genes17101284/s1. Supplementary Table S1. Clinical, demographic, and molecular characteristics of the probands and available family members included in this study. This table summarizes individual-level clinical, demographic, and chromosomal microarray analysis (CMA) data for all probands and available family members evaluated in the study. Variables include family and individual identifiers, relationship to the proband, sex, prenatal/postnatal status, age or gestational age at testing, CNV type, chromosomal coordinates, CNV size, cytoband, segment classification (proximal, distal, or spanning both regions), copy number state, pathogenicity classification, key genes within the CNV interval, parental testing status, inheritance pattern, and clinical indications. The detected CNVs column lists all array findings per individual, including the 1q21.1 CNV and any additional variants identified; only CNVs meeting minimum quality criteria (≥20 markers/probes) were included. All additional CNVs detected outside the 1q21.1 region were classified as benign or likely benign. The apparent Xp22.33q28 gain initially reported in P019 was excluded by FISH as an array artifact. Secondary referral indications denote additional clinical findings recorded at the time of referral. All CNVs were identified by CMA using the CytoScan HD Array platform (Affymetrix/Thermo Fisher Scientific) and interpreted based on the GRCh38/hg38 reference genome. P011 is shown in two rows (CNV1 and CNV2) because she carried two coexisting 1q21.1 CNVs, a proximal duplication and a distal deletion, both inherited from the father. Parental samples were unavailable for three probands, for whom parental testing is indicated as “No.” For P007, paternal CMA had been performed at an external center and was reported as normal; the father is therefore not listed. CMA, chromosomal microarray analysis; CNV, copy number variation; ACMG, American College of Medical Genetics and Genomics; ClinGen, Clinical Genome Resource. Breakpoint class was inferred from the overlap of each CNV with the ClinGen-curated proximal 1q21.1 TAR region (BP1–BP3/BP2–BP3; ISCA-37428; chr1:145,686,999–146,048,495; haploinsufficiency score 0, triplosensitivity score 1) and distal 1q21.1 recurrent region (BP3–BP4; ISCA-37421; chr1:147,105,904–147,917,509; haploinsufficiency score 3, triplosensitivity score 2). Each CNV, including the two CNVs in P011, was classified independently using the ACMG/ClinGen point-based framework [15]; evidence codes, points, and total scores are given in the proband rows, and parental rows show the classification of the same CNV. Thresholds: pathogenic, ≥0.99; likely pathogenic, 0.90–0.98; uncertain significance, −0.89 to 0.89; likely benign, −0.90 to −0.98; benign, ≤−0.99. Because the TAR region is not curated as haploinsufficient and RBM8A is associated with an autosomal recessive disorder, the proximal deletions in P003 and P008 were classified as pathogenic null alleles of RBM8A in the context of autosomal recessive TAR syndrome; their dosage-based scores (0.00) are shown for completeness. The clinical findings in P003 and P008 were considered potentially compatible with TAR syndrome, which could not be molecularly confirmed because the second RBM8A allele could not be investigated. AR, autosomal recessive; HI, haploinsufficiency; TS, triplosensitivity; TAR, thrombocytopenia-absent radius.
